# The siRNA-mediated knockdown of GluN3A in 46C-derived neural stem cells affects mRNA expression levels of neural genes, including known iGluR interactors

**DOI:** 10.1371/journal.pone.0192242

**Published:** 2018-02-13

**Authors:** Svenja Pachernegg, Sebastian Eilebrecht, Elke Eilebrecht, Hendrik Schöneborn, Sebastian Neumann, Arndt G. Benecke, Michael Hollmann

**Affiliations:** 1 The Florey Institute of Neuroscience and Mental Health, Parkville, Australia; 2 Department of Biochemistry I - Receptor Biochemistry, Ruhr University Bochum, Bochum, Germany; 3 International Graduate School of Neuroscience, Ruhr University Bochum, Bochum, Germany; 4 Ruhr University Research School, Ruhr University Bochum, Bochum, Germany; 5 CNRS UMR8246, Université Pierre et Marie Curie, Paris, France; 6 German Cancer Research Center, Heidelberg, Germany; 7 Fraunhofer Institute for Molecular Biology and Applied Ecology, Schmallenberg, Germany; 8 Department of Biochemistry II - Molecular Neurobiochemistry, Ruhr University Bochum, Bochum, Germany; 9 Department of Biochemistry II - Molecular Biochemistry, Ruhr University Bochum, Bochum, Germany; 10 Center for Innate Immunity and Immune Disease, University of Washington School of Medicine, Seattle, Washington, United States of America; Universidad de Castilla-La Mancha, SPAIN

## Abstract

For years, GluN3A was solely considered to be a dominant-negative modulator of NMDARs, since its incorporation into receptors alters hallmark features of conventional NMDARs composed of GluN1/GluN2 subunits. Only recently, increasing evidence has accumulated that GluN3A plays a more diversified role. It is considered to be critically involved in the maturation of glutamatergic synapses, and it might act as a molecular brake to prevent premature synaptic strengthening. Its expression pattern supports a putative role during neural development, since GluN3A is predominantly expressed in early pre- and postnatal stages. In this study, we used RNA interference to efficiently knock down GluN3A in 46C-derived neural stem cells (NSCs) both at the mRNA and at the protein level. Global gene expression profiling upon GluN3A knockdown revealed significantly altered expression of a multitude of neural genes, including genes encoding small GTPases, retinal proteins, and cytoskeletal proteins, some of which have been previously shown to interact with GluN3A or other iGluR subunits. Canonical pathway enrichment studies point at important roles of GluN3A affecting key cellular pathways involved in cell growth, proliferation, motility, and survival, such as the mTOR pathway. This study for the first time provides insights into transcriptome changes upon the specific knockdown of an NMDAR subunit in NSCs, which may help to identify additional functions and downstream pathways of GluN3A and GluN3A-containing NMDARs.

## Introduction

Ever since its discovery in 1995, the N-methyl-D-aspartate receptor (NMDAR) subunit GluN3A was considered to be a dominant-negative regulator of NMDARs by abolishing their Mg^2+^ block and by reducing their Ca^2+^ permeability and current responses [1–5]. Consequently, it was generally assumed that GluN3A has a neuroprotective function by decreasing glutamate-induced excitotoxicity [6–9]. Recently, evidence for a more diverse role of the GluN3 subunits than simply being down-regulators of NMDAR function has accumulated. GluN3 was suggested to support the developmental switch from GluN2B and GluN2D (prenatally) to GluN2A and GluN2C subunits (postnatally) [10, 11] via the interaction with PACSIN1 (protein kinase C and casein kinase substrate in neurons protein 1), which is involved in clathrin-mediated endocytosis and actin rearrangement [12]. Immature GluN1/GluN2B/GluN3A triheteromers are rapidly removed from glutamatergic synapses, undergoing endocytosis and transport to early endosomes, a process which relies on the interaction of GluN3A with PACSIN1 [12]. GluN3A undergoes clathrin-mediated endocytosis also through binding to the clathrin adaptor complex AP2 [13]. Recently, it was suggested that the incomplete removal of juvenile GluN3A-containing NMDARs might contribute to the pathophysiology of Huntington’s disease [14, 15].

Findings in GluN3 mouse models support an involvement of GluN3 subunits in the proper maturation of glutamatergic synapses. GluN3A-overexpressing mice are severely impaired both in learning and long-term memory storage and show reduced hippocampal LTP [16]. Moreover, the number and size of synapses in these mice are decreased, as is the density of dendritic spines [16]. Consistent with these findings, in GluN3A knockout (KO) mice, dendritic spine density is increased [2] and glutamatergic synapses mature more rapidly [17]. Thus, GluN3A might act as a molecular brake, which inhibits the premature strenghtening of glutamatergic synapses [16–18].

In this study, we aimed to further elaborate the role of GluN3A during neural development. To this end, we used the 46C embryonic stem cell (ESC) system. This murine stem cell line was generated by cloning the coding sequence (CDS) of eGFP as well as a puromycin resistance gene under control of the Sox1 promoter in E14Tg2a.IV cells [19, 20]. Since Sox1 is the earliest known neuroectodermal marker [21], the cells fluoresce greenly as soon as they are differentiated into neuroepithelial precursor cells (NEPs), which express Sox1. In turn, NEPs can be differentiated either into neurons via treatment with retinoic acid (RA), or into radial glia-like neural stem cells (NSCs) via prolonged cultivation in the neuroinductive medium N2B27 supplemented with basic fibroblast growth factor (bFGF) and epidermal growth factor (EGF) [22–24]. 46C-derived NSCs can then be differentiated into astrocytes via the addition of fetal calf serum (FCS) [23, 25]. We and others have shown that 46C ESCs and their derivatives express the appropriate stem cell and differentiation markers [20, 22–26]. In this study, the expression of GluN3A in 46C-derived cells was determined via quantitative real time PCRs (qRT-PCRs) and Western blots. Next, an siRNA approach was used to knock down GluN3A in 46C-derived NSCs, and the knockdown was confirmed both at the mRNA and protein levels. Finally, global gene expression profiling was performed to examine the effect of GluN3A knockdown on gene expression. Besides a variety of pathways involved in cell growth, proliferation, motility, and survival being significantly affected by the GluN3A knockdown, we found that the mRNA expression of several neural genes is affected by the knockdown of GluN3A. Some of the corresponding gene products have been previously shown to interact with NMDARs or other iGluR subunits. Although we have previously shown that 46C-derived NSCs do not express functional NMDARs [27], the NSCs served as an *in vitro*-system to investigate the transcriptome changes in neural cells after knockdown of GluN3A. The detected up- and downregulated genes can serve as guidance for researchers interested in putative NMDAR-regulated proteins.

## Materials and methods

### Synthetic siRNAs against GluN3A

The CDS of GluN3A (*Mus musculus*; Accession No: NM_001276355) was submitted to the siRNA services of various companies (Clontech, Eurogentec, GE Dharmacon, Thermo Fisher Scientific Ambion, and Invitrogen) to receive putative siRNA sequences directed against GluN3A. Using a scoring system for siRNAs [28], the two highest scoring siRNA sequences were identified and two scrambled siRNAs were designed (www.siRNAwizard.com/scrambled.php) as negative controls. All sequences were checked if they match any murine mRNA other than that of GluN3A using BLAST (Basic Local Alignment Search Tool; blast.ncbi.nlm.nih.gov./Blast.cgi), which they did not. The four RNA sequences (siRNA1, siRNA2, scrambled siRNA1, and scrambled siRNA2; Table 1) were synthesized by Microsynth.

**Table 1:** Synthetic siRNA and scrambled siRNA sequences.

	Sequence (5’→3’)
siRNA1	cacccacaauggugauguutt
scrambled siRNA1	gacaguaccgcauucuagutt
siRNA2	gaagaaugauccagagaaatt
scrambled siRNA2	gaggcaacaacaauagauatt

### Cell culture

46C ESCs were kindly provided by Prof. Austin Smith (University of Cambridge, UK). All 46C-derived cell cultures were maintained at 37 °C and 5% CO_2_. 46C ESCs were cultured in GMEM containing 10% FCS, 10% tryptose phosphate, 0.1 mM 2-mercaptoethanol, 1.8 mM glutamine, and 1000 U/ml leukemia inhibitory factor (LIF; Merck Millipore). NEPs were differentiated from 46C ESCs by incubation in a neuroinductive medium (N2B27) as described previously [20]. NSCs were differentiated from NEPs by the prolonged cultivation in N2B27 medium and the addition of EGF and bFGF (both 10 ng/ml; Preprotech) [22]. NEPs were differentiated into neurons via the treatment with RA (10 *μ*M) for 10 days, and NSCs were differentiated into astrocytes by adding 5% FCS to the medium for 14 days. Cells were allowed to grow for 2 days before RNA or protein isolation.

### Transfection of 46C-derived NSCs

To evaluate the most efficient transfection method for 46C-derived NSCs, cells were plated on 3.5 cm cell culture dishes and transfected with an eGFP plasmid. The following lipofection reagents were compared: Arrest-In (Open Biosystems), FuGene HD (Roche), Gene Juice (Novagen), LipoD293 (SignaGen Laboratories), Lipofectamine Plus (Invitrogen), Metafectene Easy (Biontex), Metafectene Pro (Biontex), TransIt Neural (Mirus), and X-tremeGENE siRNA (Roche) (Table 2). If not explicitly demanded otherwise by the manufacturer’s instructions, 46C-derived NSCs were transfected 4 hours after passaging. Cytotoxicity and transfection rate were analyzed 48 hours after transfection by counting the number of dead and transfected cells, respectively. Dead cells were identified by Trypan Blue (Sigma Aldrich) staining, transfected cells by their green fluorescence. Afterwards the percentage of both dead and transfected cells was calculated.

**Table 2:** Transfection conditions of different lipofection reagents. 46C-derived NSCs were plated in 3.5 cm cell culture dishes.

Lipofection reagent	eGFP plasmid DNA	Hours (after passaging)	Cell density
10 *μ*l Arrest-In	2 *μ*g	24	2x10^5^
6 *μ*l FuGene HD	3 *μ*g	4	1x10^5^
3 *μ*l Gene Juice	3 *μ*g	24	2x10^5^
15 *μ*l LipoD293	5 *μ*g	24	5x10^5^
4 *μ*l Lipofectamine + 16 *μ*l Plus	1.6 *μ*g	4	3x10^5^
20 *μ*l Metafectene Easy	20 *μ*g	0	7x10^5^
6 *μ*l Metafectene Pro	3 *μ*g	4	3x10^5^
10 *μ*l TransIt Neural	2.5 *μ*g	24	3x10^5^
60 *μ*l X-tremeGENE siRNA	12 *μ*g	24	1x10^5^

For the lipofection of 46C-derived NSCs with GluN1-1a-peGFP-N1, 1x10^6^ cells were plated on 10 cm cell culture dishes 4 hours prior to transfection and transfected with 10 *μ* plasmid DNA and 20 *μ*l FuGene HD (Roche).

For the lipofection of 46C-derived NSCs with synthetic siRNAs, X-tremeGENE siRNA (Roche) was used. To this end, 1x10^5^ 46C-derived NSCs were plated on 3.5 cm cell culture dishes 24 hours prior to transfection and transfected with 12 *μ*g siRNA and 60 *μ*l X-tremeGENE siRNA (Roche).

### Reverse transcription and quantitative real time PCR

Total RNA was isolated from cell cultures and control tissue (mouse whole brain from postnatal day 3; P3) using the GenElute Mammalian Total RNA Mini-Kit (Sigma-Aldrich) or NucleoSpin RNA Kit (Macherey-Nagel). Prior to RNA isolation, cells were gently washed with 1 X PBS to remove dead cells and residual medium. 2 ml of 1 X PBS were added to the cell culture dishes, and the cells were dissociated mechanically from the cell culture vessels with sterile cell scrapers. The cell suspension was then briefly centrifuged for 5 min at 1000 rpm to pellet the cells. Whole brain RNA of neonatal (P3) C57BL/6 mice served as positive control. Frozen whole mouse brains (P3) were kindly provided by the laboratory of Prof. Hanns Hatt (Ruhr University Bochum, Germany). All animal procedures were performed in accordance with the German Animal Welfare Act. Brains were dissected into hemispheres; a single hemisphere was cut into small pieces with a scalpel and suspended in 2 ml of 1 X PBS. The following steps of RNA isolation for both cells and control tissue were performed following the manufacturer’s instructions. The total RNA isolation procedure included a DNase I (Promega) treatment on the purification column according to the manufacturer’s protocol. To generate cDNA, 2 *μ*g of RNA per sample was reverse-transcribed by SuperScriptII Reverse Transcriptase (Thermo Fisher Scientific Invitrogen) using random hexamer primers (Promega). Subsequently, 50 ng of cDNA per sample were used as template in qRT-PCRs on the Roche Light Cycler 2.0 using the FastStart DNA Master^*PLUS*^ SYBR Green I Kit (both Roche Diagnostics) or the QuantiTect SYBR Green RT-PCR Kit (Qiagen) according to the manufacturers’ instructions. For primer sequences, see Table 3. qRT-PCR results were analyzed using Roche LightCycler Software 3.5 (Roche Diagnostics).

**Table 3:** Primers used in qRT-PCRs.

Gen	Sequence 5’→3’
GluN1	gctgtacctgctggaccgct
	gcagtgtaggaagccacgatgatc
GluN2A	gctacgggcagacagagaag
	gtggttgtcatctggctcac
GluN2B	gctacaacacccacgagaagag
	gagagggtccacactttcc
GluN2C	aaccacaccttcagcagcg
	gacttcttgcccttggtgag
GluN2D	cgatggcgtctggaatgg
	agatgaaaactgtgacggcg
GluN3A	gatggagctggacttggtca
	ccagttgttcatggtcaggat
GLAST	ccaaagcaacgagaagagc
	ctccccagggaacgaaaagta
Pax6	agttcttcgcaacctggcta
	cccgggcaaacacatctgga
Prominin	gacactccctatctgctcaag
	gctcttccttcctgtgttatc
Nestin	ggaccggttgcagcccactga
	ggcaagggggaagagaaggatgt
*β*-actin	cgttgacatccgtaaagacct
	caaagccatgccaatgttgtctct
GAPDH	catcaacgaccccttcatt
	ctccacgacatactcagcac
Ubiquitin	ctgggcggttgcttt
	ggttgactccttctggatgtt

The qRT-PCR reaction was performed with an initial pre-incubation for 10 min at 95 °C, followed by 40 amplification cycles (95 °C for 10 s, 59 °C for 10 s, and 72 °C for 20 s). In addition, melting curve analysis was performed to analyse the specificity of the primers and the amplified DNA fragment. For this, at the end of each qRT-PCR run, the temperature was raised from 65 °C to 95 °C at a rate of 0.1 °C/s and the fluorescence in the samples was measured continously. Roche LightCycler Software 3.5 (Roche Diagnostics) was used to calculate the melting point.

Quantitative real time data was obtained by mathematical modeling [29]. The expression of the genes of interest was normalized to the mean expression of the three housekeeping genes *β*-actin, GAPDH, and ubiquitin (2^Δ*Ct*^ method). To additionally compare the expression of genes in different 46C-derived cell types to their expression in a control, the data was also normalized to the expression of genes in mouse whole brain (2^ΔΔ*Ct*^ method). Statistics were calculated using Prism 5.0 software (GraphPad).

### Transcriptome analysis

Total RNA was isolated from the cells using the GenElute Mammalian Total RNA Mini Kit (Sigma-Aldrich), and 2 *μ*g of RNA were used in reverse transcriptions to generate cDNA. Subsequently, cRNA was generated from cDNA and labelled with digoxigenin (DIG) in an *in vitro* transcription reaction using the RT Labeling Kit (Thermo Fisher Scientific Applied Biosystems). The cRNA was then hybridized to AB1700 murine microarray chips and detected following the manufacturer’s protocol (Thermo Fisher Scientific Applied Biosystems). With an AB1700 Chemiluminescent Microarray Analyzer (Thermo Fisher Scientific Applied Biosystems), the expression of 32 966 validated murine genes was analyzed. For each condition (cells transfected with either a mixture of GluN3A siRNA1/GluN3A siRNA2 or with a mixture of scrambled siRNA1/scrambled siRNA2), two different batches of 46C-derived NSCs were transfected to measure two biological replicates; for each biological replicate two technical replicates were measured as well, totaling 4 replicates per condition (2 technical replicates x 2 biological replicates). The microarray data were extracted and median-normalized. Data quality was determined using a QC procedure [30]. Data were normalized using NeONORM with k = 0.02 [31–33]. Subtraction profiling was performed on merged technical replicates for each biological replicate as described previously [34, 35] using the CDS test [36]. Differentially expressed genes were classified using Ingenuity Pathway Analysis software to detect network and pathway enrichments. Transcriptome data were deposited in the public database MACE (http://mace.ihes.fr) using Accession No: 3109613596.

### Protein biochemistry

Total membrane proteins of cell cultures and control tissue (whole brain from P3 C57BL/6 mice; kindly provided by the laboratory of Prof. Hanns Hatt, Ruhr University Bochum, Germany) were prepared by hypotonic lysis. To this end, cells were gently washed with 1 X PBS to remove dead cells and residual medium. 2 ml of 1 X PBS were added to the cell culture dishes, and the cells were dissociated mechanically from the cell culture vessels with sterile cell scrapers. The cell suspension was then briefly centrifuged for 5 min at 1000 rpm to pellet the cells. Frozen whole mouse brains (P3) were dissected into hemispheres, and a single hemisphere was cut into small pieces with a scalpel and suspended in 2 ml of 1 X PBS. Cells and tissues were incubated in lysis buffer (10 mM HEPES/KOH, 1.5 mM KCl, 10 mM MgCl_2_, 0.5 mM DTT) for 15 min at 4 °C. Subsequently, the samples were transferred into a douncer and the cell membranes were disrupted by approximately 20 strokes with a loose-fitting pestle. The homogenous cell solution was then centrifuged for 10 min at 2000 rpm at 4 °C, and the supernatant was ultracentrifuged to pellet membrane-bound proteins (100 000 x g; 1 h). The membrane protein pellet was resuspended in 2 X Laemmli buffer with urea such that 25 *μ*l buffer was used per 1x10^6^ cells. The cell number was determined by counting an aliquot of the cell nuclei in a Neubauer counting chamber.

Plasma membrane proteins of cell cultures were isolated using biotinylated concanavalin A (10 *μ*M; Sigma-Aldrich) followed by ultracentrifugation (100 000 x g; 1 h). A sample of the supernatant, which contains total protein, was collected prior to the following precipitation. The remaining supernatant was precipitated with streptavidin-agarose beads (Fluka), incubated for 3 hrs and again pelleted by a 2-min spin at 13 000 x g. The final pellets were boiled in Laemmli buffer (containing urea) for 10 min to disrupt protein binding to the beads.

For SDS-PAGE (7.5% acrylamide), 50 *μ*g of membrane proteins were loaded per sample. After transferring the proteins to nitrocellulose membranes, the membranes were reversibly stained with Ponceau S (0.2% Ponceau S, 3% trichloroacetic acid, 3% sulfosalicylic acid) to check for the proper transfer of proteins onto the membrane. For immunoblot analysis, unspecific binding sites of the nitrocellulose membranes were blocked by incubation for one hour in blocking solution (5% dry milk powder w/v in PBS-T) at room temperature and under constant agitation. The membranes were then washed twice for 5 min each with 1 X PBS-T and subsequently incubated with the appropiate primary antibody (1 h at room temperature under constant agitation). The following primary antibodies were used: anti-calnexin (1:500 in 1 X PBS-T; Santa Cruz Biotechnology), anti-GluN3A (1:1000 in 1 X PBS-T; Merck Millipore Upstate), and anti-GluN1 (1:1000 in in 1 X PBS-T; Merck Millipore Upstate). For detection, appropriate HRP-conjugated secondary antibodies (1:10 000 in blocking solution; Sigma-Aldrich) were used. For the removal of antibodies from previously probed Western blots to allow reprobing of the membranes with another antibody, the membranes were incubated for 2 h in stripping buffer (25 mM glycine; pH 2.0, 1% SDS). Densitometric analysis of Western blots was performed with the Fiji/ImageJ 1.51n software [37, 38].

For immuncytochemical stainings, 46C-derived NSCs were briefly washed twice with PBS/A (PBS + 0.1% (w/v) BSA). The cells were fixed with 4% (w/v) PFA for 10 min at room temperature and incubated with the primary antibody diluted in PBT1 (PBS + 1.0%(w/v) BSA + 0.1% (v/v) Triton X-100) at RT for 30 min. The following primary antibody was used: anti-GluN3A (1:100; Santa Cruz Biotechnology). After incubation with the primary antibody, the incubation with the Cy3-coupled species-specific secondary antibody (1:500; Dianova) diluted in PBS/A was performed at RT for 30 min. Finally, the nuclei were stained with Hoechst 33342 (1 *μ*g/ml; Thermo Fisher Scientific) and mounted in PBS/glycerol (2:1). Pictures were taken at an Axioplan2 microscope with the AxioCam HRc camera using the AxioVision 4.4 and 4.5 software (Zeiss).

## Results

### Expression of GluN3A in 46C-derived NSCs

**Fig 1:**
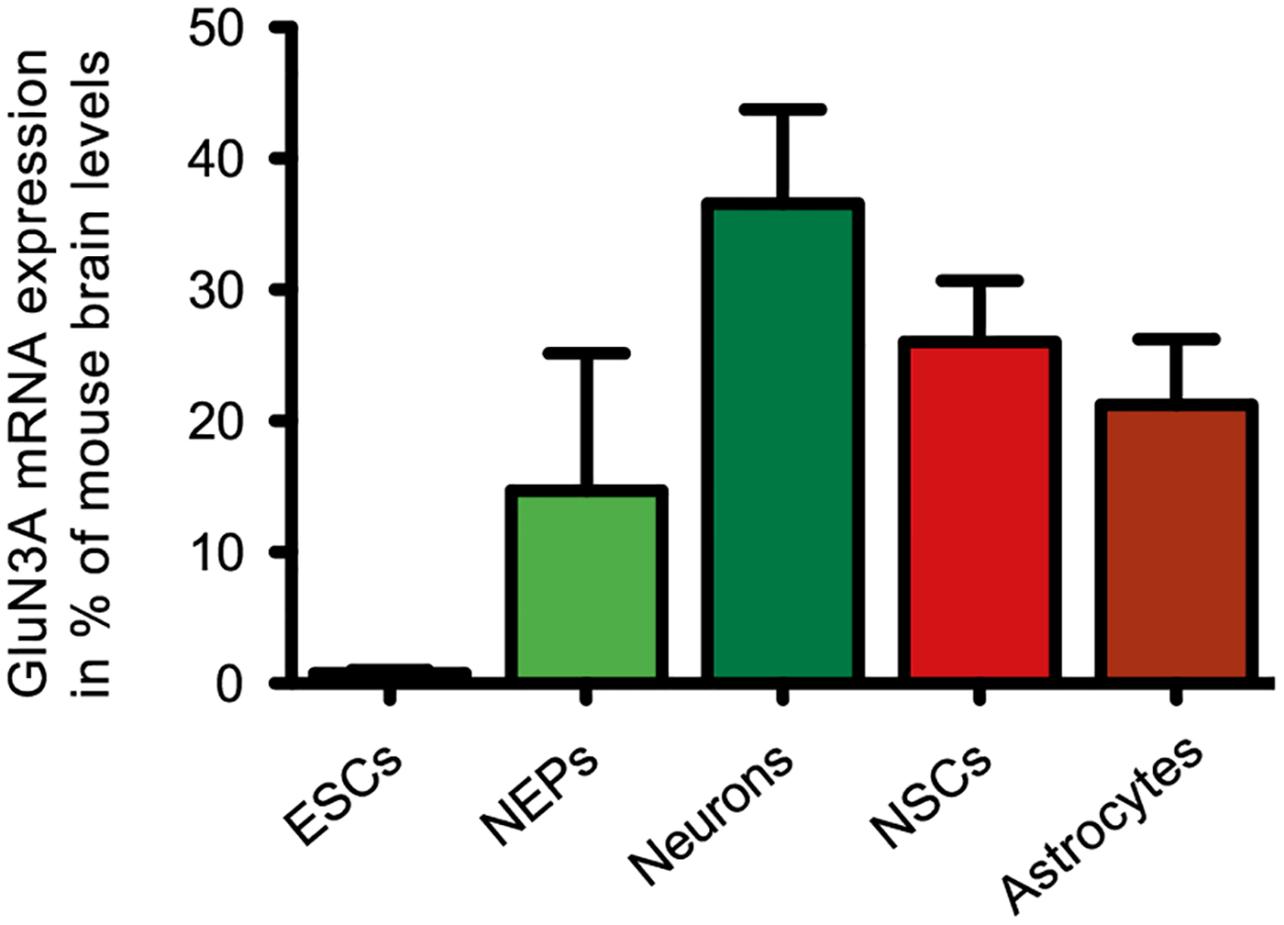
Expression of GluN3A mRNA in 46C-derived cells. GluN3A was expressed in all 46C-derived cells at the mRNA level and its transcript expression in 46C-derived NSCs amounted to 26% of its expression in mouse whole brain. Data represent means +/- SEM; there were no statistically significant differences in GluN3A expression between either of the depicted cell types as tested by one-way ANOVA followed by Tukey’s multiple comparison test; n = 3 (ESCs, NEPs, and astrocytes), 6 (neurons), or 21 independent experiments (NSCs).

**Fig 2:**
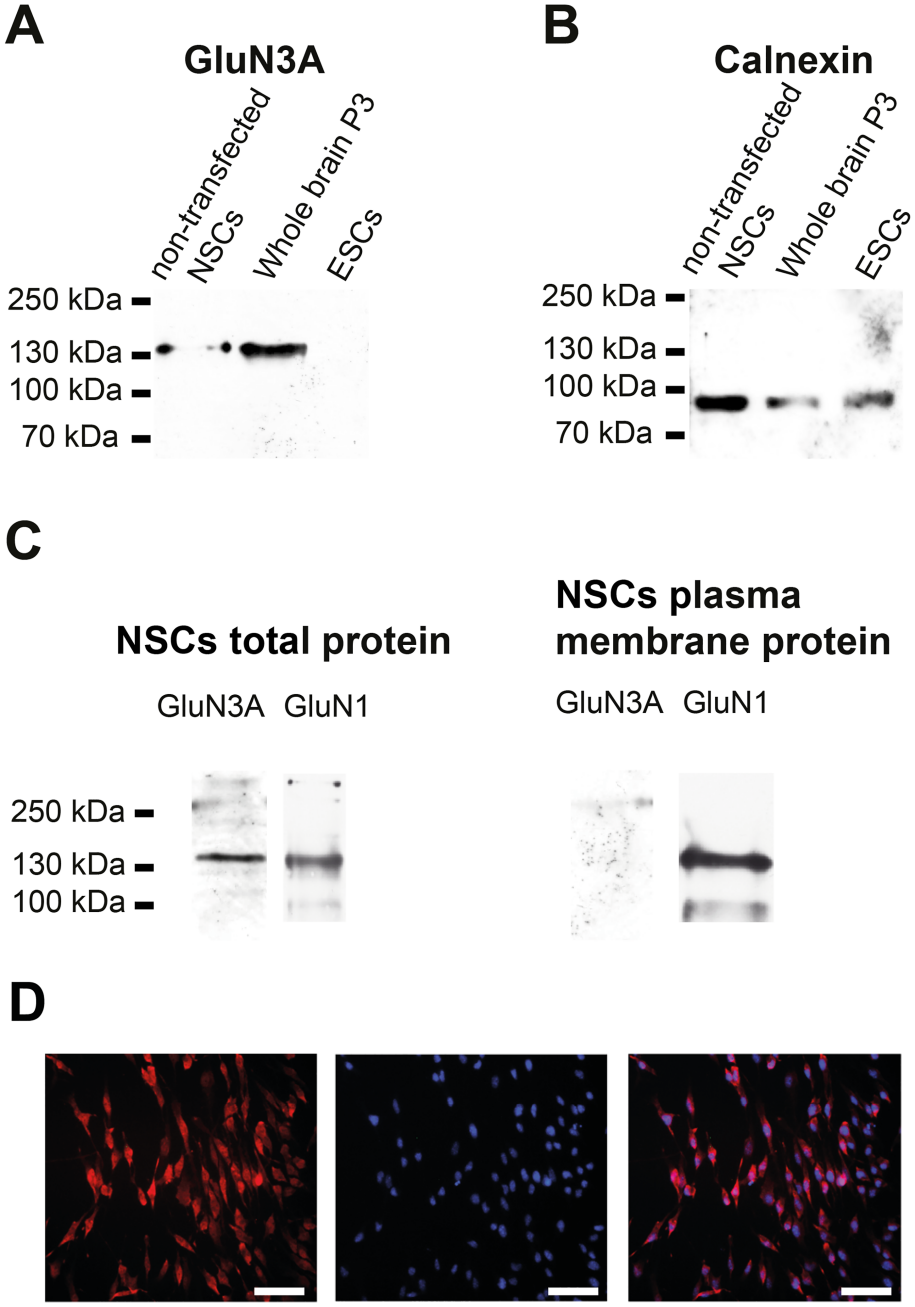
Protein expression of GluN3A in whole brain, 46C ESCs, non-transfected NSCs, and GluN1-transfected NSCs. **A:** GluN3A expression in total protein preparations. Bands at the expected molecular weight of GluN3A (130 kDa) were visible in the positive control (mouse whole brain P3) and in non-transfected 46C-derived NSCs. Undifferentiated 46C ESCs did not express GluN3A proteins. **B:** Expression of the housekeeping protein calnexin (90 kDa) as a control. **C:** GluN3A expression in total and plasma membrane protein preparations from GluN1-transfected 46C-derived NSCs. GluN3A protein expression was not detectable in the plasma membrane fraction of 46C-derived NSCs. GluN1 (130 kDA) surface expression in GluN1-transfected 46C-derived NSCs in the same total and plasma membrane protein preparations is shown to demonstrate that the biotinylated concanavalin A-treatment of NSCs successfully enriched plasma membrane protein. **D:** 46C-derived NSCs were immuncytochemically stained for GluN3A (red fluorescence), and cell nuclei were stained with Hoechst 33342 (blue fluorescence). The staining confirmed the expression of GluN3A in the cytoplasm of 46C-derived NSCs. Scale bars: 50 *μ*m.

First, the mRNA expression of GluN3A in different 46C-derived cell types (ESCs, NEPs, NSCs, neurons, and astrocytes) was investigated by qRT-PCRs (Fig 1). GluN3A transcripts were expressed in all 46C-derived cell types, even weakly in undifferentiated ESCs (approximately 0.8% compared to its expression in mouse whole brain). Notably, the transcript expression of GluN3A in NSCs was approximately 70% of that observed in early neurons differentiated from 46C-derived NEPs and 26% compared to its expression in mouse whole brain P3. To investigate GluN3A protein expression in 46C-derived NSCs, total membrane fractions of NSCs were analyzed via Western blot. GluN3A is expressed at the protein level in 46C-derived NSCs and in the positive control (protein isolated from mouse whole brain P3), but not in undifferentiated 46C ESCs (Fig 2A). Additionally, the plasma membrane expression of GluN3A was investigated in GluN1-transfected 46C-derived NSCs (Fig 2C). GluN3A protein expression was detected in total protein isolated from NSCs, but not in NSC plasma membrane protein. GluN1 surface expression in the same plasma membrane protein preparations of GluN1-transfected 46C-derived NSCs is shown to demonstrate that the biotinylated concanavalin A-treatment of NSCs successfully enriched plasma membrane proteins. Immuncytochemical stainings of 46C-derived NSCs for GluN3A confirmed its expression in the cytoplasm (Fig 2D).

### Synthetic siRNA-mediated knockdown of GluN3A in 46C-derived NSCs

**Fig 3:**
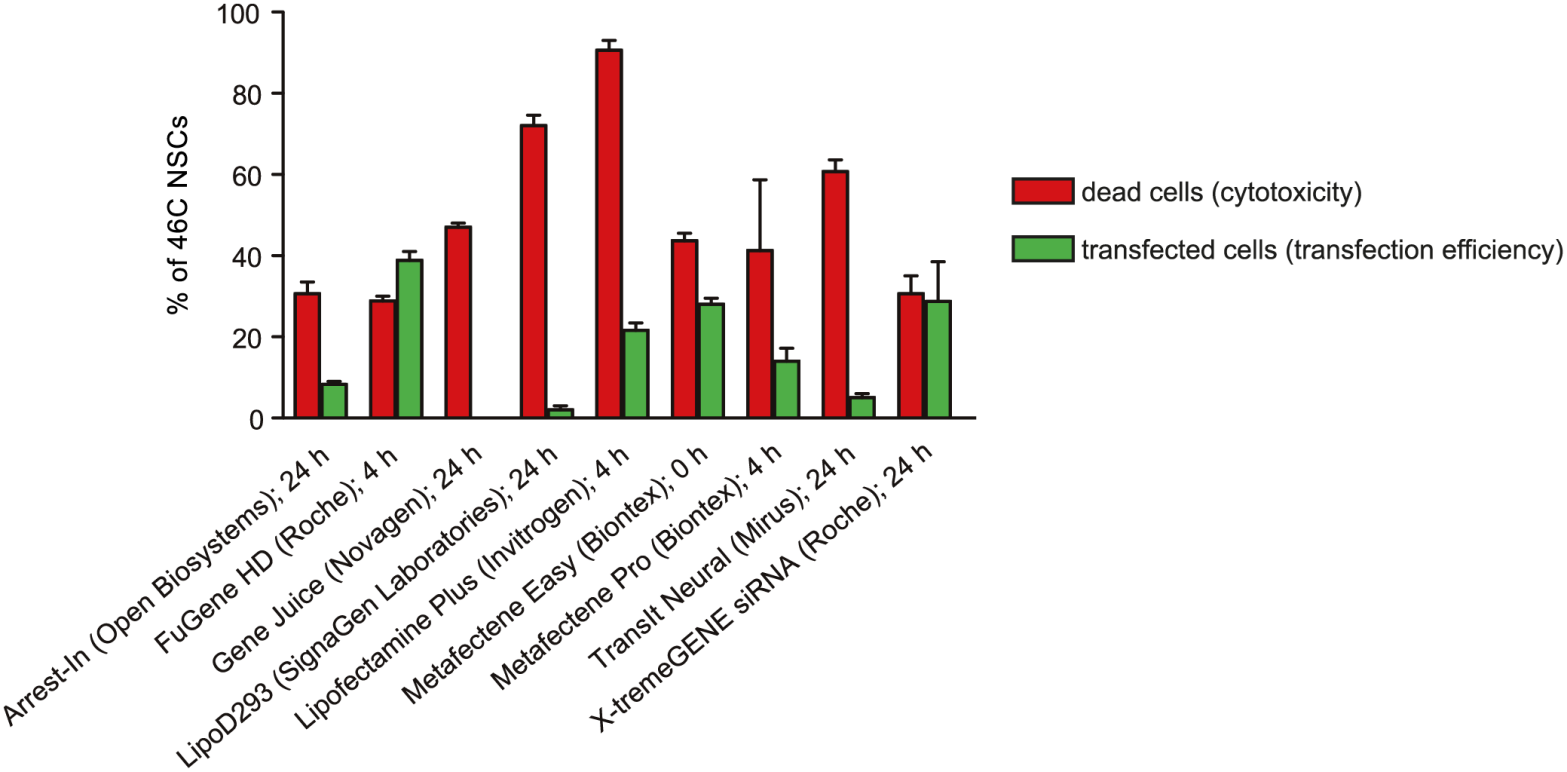
Cytotoxicity and transfection rate of various lipofection reagents for 46C-derived NSCs. 46C-derived NSCs were transfected with an eGFP plasmid using various lipofection reagents. If the corresponding transfection protocol allowed to freely choose the transfection time point, NSCs were transfected 4 hrs after passaging. Otherwise, cells were transfected as suggested by the reagent’s manual (0 h or 24 hrs after passaging). X-tremeGENE siRNA (Roche) was used to transfect 46C-derived NSCs with synthetic siRNAs in the following experiments.

In order to target as many cells as possible, the most efficient transfection method for 46C-derived NSCs was determined beforehand by transfecting an eGFP plasmid using various lipofection reagents. Whenever possible, 46C-derived NSCs were transfected 4 hrs after passaging, which was previously determined to be the best transfection time point for 46C-derived NSCs. However, some lipofection reagents required to be used at different time points (0 h after passaging, or 24 hrs after passaging). For the transfection with the synthetic siRNAs, we used X-tremeGENE siRNA (Roche), which was specifically developed to deliver synthetic siRNAs into cells and yielded satisfying transfection results in the transfection experiments with the eGFP plasmid (Fig 3). Since the synthetic siRNAs are considerably smaller than the eGFP plasmid, it can reasonably be assumed that the transfection rate with X-tremeGENE siRNA was at least comparable to the eGFP plasmid control. Moreover, the transfection rate of siRNAs may even be significantly higher than the transfection rate of the eGFP plasmid, as suggested by the strength of knockdown of the target mRNA (see below).

**Fig 4:**
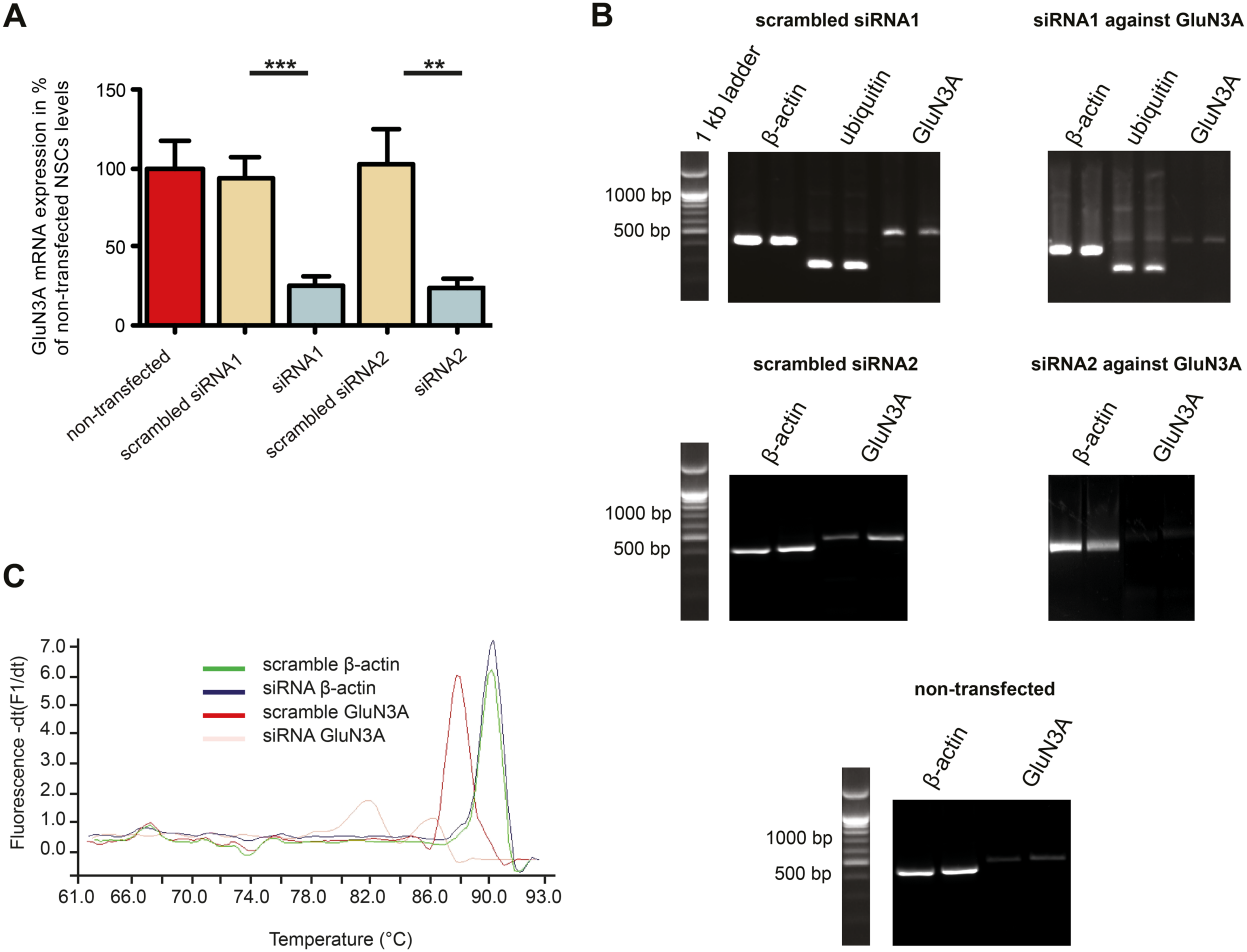
siRNA-mediated knockdown of GluN3A mRNA in 46C-derived NSCs. 46C-derived NSCs were either transfected with scrambled siRNA or siRNA directed against GluN3A. **A:** qRT-PCRs were performed to analyse the knockdown of GluN3A at the mRNA level. GluN3A was significantly downregulated upon transfection with siRNA against GluN3A. There were no statistically significant differences in GluN3A expression between non-transfected NSCs and NSCs transfected with either scrambled siRNA1 or scrambled siRNA2. **B:** Agarose gel electrophoresis after qRT-PCR. The band of GluN3A is much weaker in 46C-derived NSCs transfected with siRNA1 or siRNA2 directed against GluN3A than in 46C-derived NSCs transfected with scrambled siRNA1 or scrambled siRNA2, respectively. Non-transfected 46C-derived NSCs show weak GluN3A expression as well. Expected fragment sizes: 368 bp (*β*-actin), 240 bp (ubiquitin), and 417 bp (GluN3A). **C:** Melting point analysis was performed after the qRT-PCRs to determine the melting points of *β*-actin and GluN3A in transfected 46C-derived NSCs. The melting points of the GluN3A fragments differed in cDNA isolated from 46C-derived NSCs either transfected with scrambled siRNA1 or with siRNA1 directed against GluN3A, whereas the melting points of *β*-actin were identical in both NSC populations. Data represent means +/- SEM; statistical significances were assigned by unpaired Student’s *t*-test. **p < 0.01; ***p < 0.001. n = 6-9 independent experiments.

48 hours post transfection, the knockdown of GluN3A was analyzed both at the mRNA and at the protein level. To verify the knockdown of GluN3A transcripts in transfected 46C-derived NSCs, qRT-PCRs were performed (Fig 4A). While the mRNA expression of GluN3A did not differ significantly between non-transfected and scrambled siRNA-transfected 46C-derived NSCs, GluN3A transcripts were significantly downregulated in 46C-derived NSCs transfected with either siRNA1 or siRNA2 compared to 46C-derived NSCs transfected with scrambled siRNA1 or scrambled siRNA2, respectively (*p* < 0.001 and *p* < 0.01). Compared to non-transfected 46C-derived NSCs, the mRNA expression of GluN3A was decreased to approximately 24% in 46C-derived NSCs transfected with siRNA1 or siRNA2. Fig 4B shows an exemplary image taken after qRT-PCR products were separated by agarose gel electrophoresis to visualize the DNA fragments. Whereas the signals of the bands of the housekeeping genes *β*-actin and ubiquitin are of the same strength in the cDNA sample from 46C-derived NSCs transfected with scrambled siRNA1 and in the cDNA sample from 46C-derived NSCs transfected with siRNA1 directed against GluN3A, the bands of the GluN3A fragment are considerably weaker in cDNA from 46C-derived NSCs transfected with siRNA1 directed against GluN3A than in cDNA from scrambled siRNA1-transfected 46C-derived NSCs. The same holds true for the 46C-derived NSCs transfected with scrambled siRNA2 or siRNA2 directed against GluN3A, respectively. Non-transfected 46C-derived NSCs show weak GluN3A expression.

To further verify the identity of the GluN3A qPCR products, the melting points of the amplified fragments were determined after the qRT-PCRs. For this, the temperature was raised from 60 to 95 °C at a rate of 0.1 °C/s and the fluorescence in the PCR samples was measured continuously. Fig 4C shows the melting points of GluN3A and of *β*-actin, which were amplified from cDNA isolated either from 46C-derived NSCs transfected with siRNA1 directed against GluN3A or from 46C-derived NSCs transfected with scrambled siRNA1. Whereas the melting curves of the *β*-actin fragment show one single melting point at approximately 91 °C, the melting curves of GluN3A differ. In cDNA from 46C-derived NSCs transfected with scrambled siRNA1, its melting curve shows one single melting point (at approximately 88 °C), while in cDNA from 46C-derived NSCs transfected with siRNA1 directed against GluN3A, the melting curve shows two melting points: one at approximately 86 °C and one at approximately 82 °C.

**Fig 5:**
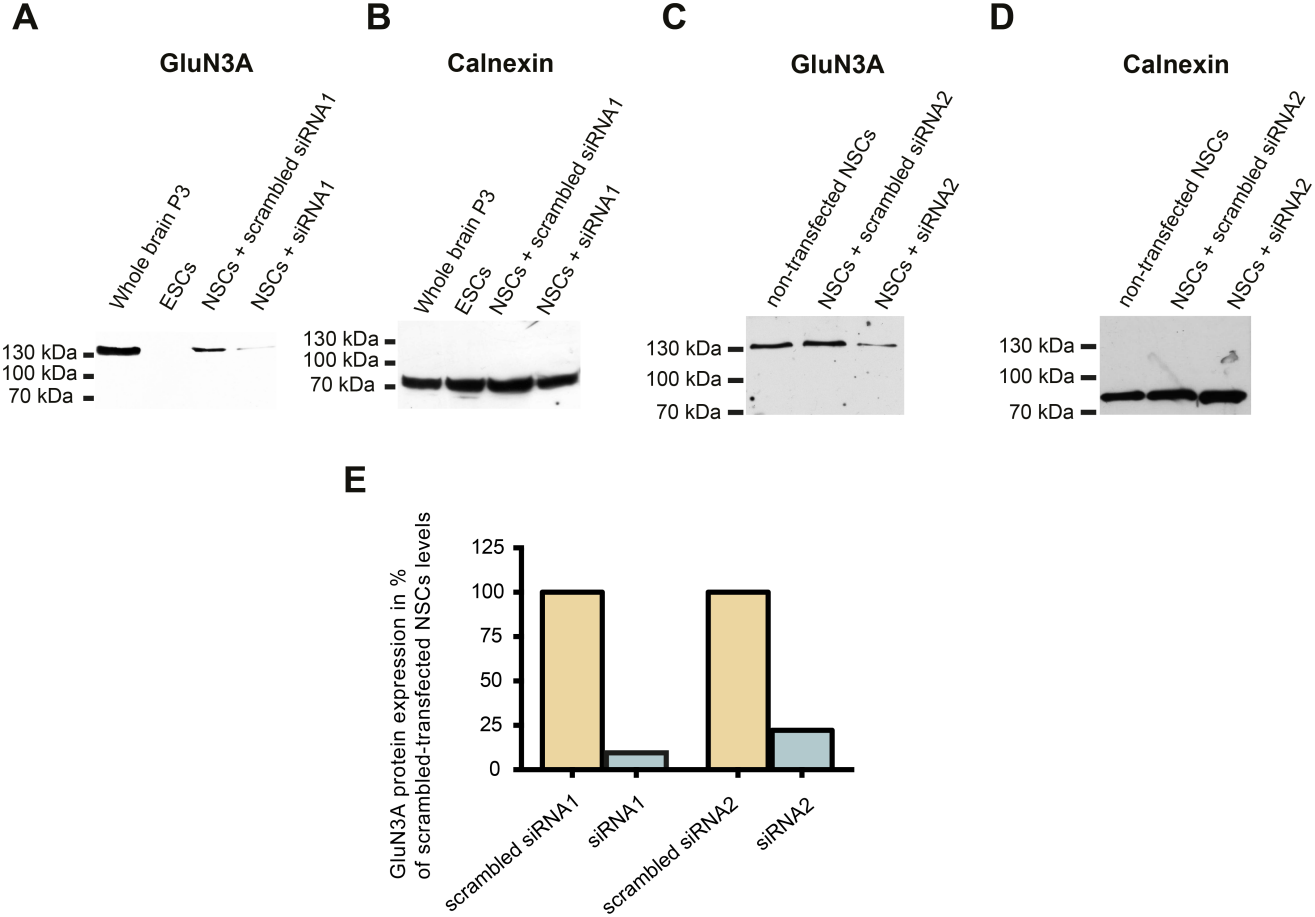
siRNA-mediated knockdown of GluN3A proteins in 46C-derived NSCs. 46C-derived NSCs were either transfected with scrambled siRNAs or with siRNAs against GluN3A. **A:** GluN3A. A strong band at the expected molecular weight for GluN3A (130 kDa) is visible in the positive control (membrane protein isolated from mouse whole brain P3), but not in the negative control (membrane protein isolated from undifferentiated 46C ESCs). In membrane protein preparations isolated from 46C-derived NSCs transfected with siRNA1 directed against GluN3A, the protein expression of GluN3A was much weaker than in 46C-derived NSCs transfected with scrambled siRNA1. **C:** The same holds true for 46C-derived NSCs transfected with siRNA2 directed against GluN3A. **B, D:** Expression of the housekeeping gene calnexin (90 kDa). Calnexin expression was not affected by siRNA transfection. **E:** Densitometric analysis of the signal intensities of GluN3A and calnexin showed that GluN3A protein expression was reduced to approximately 10% after transfection with siRNA1 and to approximately 22% after transfection with siRNA2, when compared to scrambled siRNA1- and scrambled siRNA2-transfected cells, respectively.

As the abovementioned results point to an efficient knockdown of GluN3A at the mRNA level, we next tested whether the GluN3A siRNAs also affected GluN3A protein levels via Western blot analysis (Fig 5). The Western blot signal at the expected molecular weight of GluN3A (130 kDa) was substantially weaker in membrane protein isolated from 46C-derived NSCs transfected with siRNA1 or siRNA2 directed against GluN3A when compared to membrane protein isolated from 46C-derived NSCs transfected with scrambled siRNA1 or scrambled siRNA2 (Fig 5A and 5C). Additionally, membrane protein isolated from mouse whole brain P3 and from undifferentiated 46C ESCs was applied as positive and negative control, respectively. In contrast, the Western blot signals obtained by immunodetection with an antibody directed against the housekeeping protein calnexin were equally strong in all samples (Fig 5B and 5D), thus proving that approximately the same amount of protein had been applied in all lanes. For all samples, the signal intensities for GluN3A were analyzed densitometrically and normalized to the signal intensity of calnexin. Thus, the normalized protein expression of GluN3A was determined. Additionally, the normalized protein expression of GluN3A in siRNA1- and siRNA2-transfected 46C-derived NSCs was further normalized to its expression in scrambled siRNA1- or scrambled siRNA2-transfected cells, respectively. The densitometric analysis showed that compared to the scrambled transfected cells, the protein expression of GluN3A was downregulated to approximately 10% after transfection with siRNA1 and to approximately 22% after transfection with siRNA2 (Fig 5E). In summary, the transfection of synthetic siRNAs proved to be an efficient tool to induce a knockdown of GluN3A in 46C-derived NSCs.

**Fig 6:**
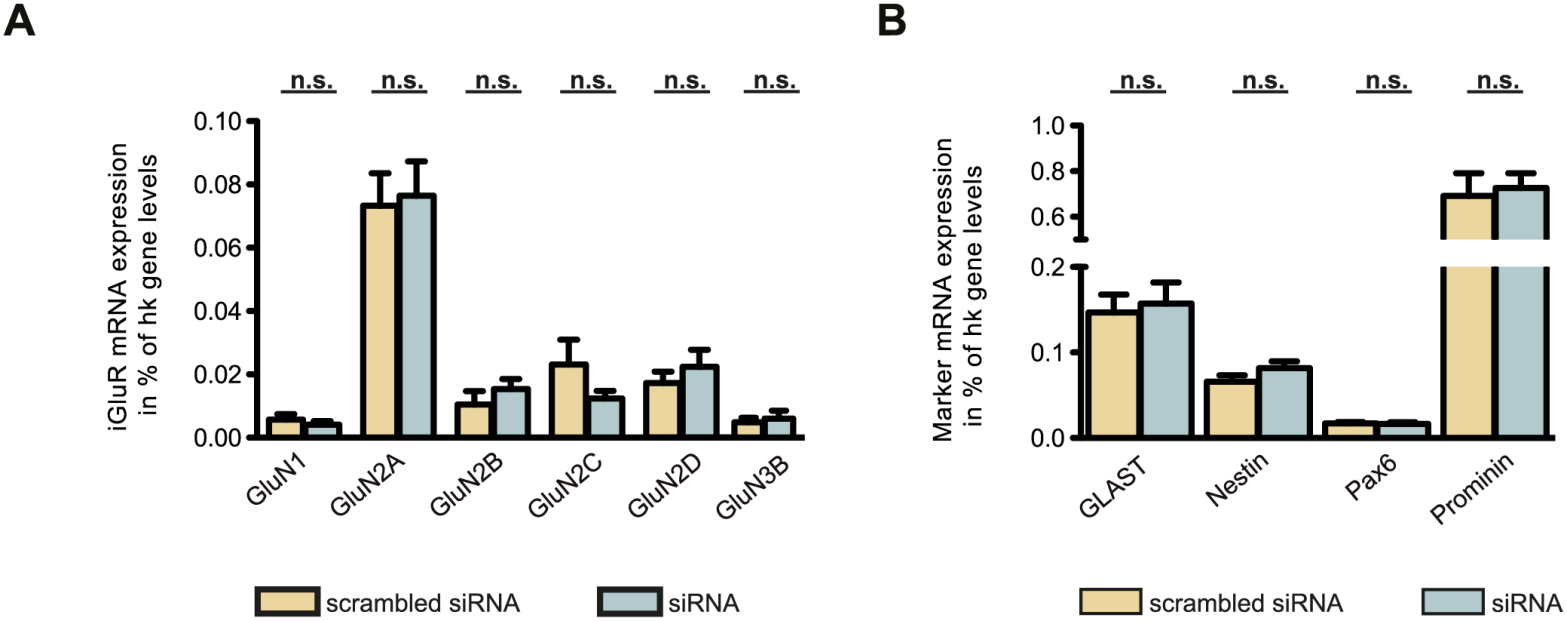
Expression of NMDAR and NSC marker mRNAs in transfected 46C-derived NSCs. **A:** NMDARs. **B:** NSC markers. 46C-derived NSCs were either transfected with a mixture of scrambled siRNA1/scrambled siRNA2 or with a mixture of GluN3A siRNA1/GluN3A siRNA2. The expression of NMDARs and NSC markers did not change significantly in 46C-derived NSCs upon transfection with siRNA against GluN3A. Data represent means +/- SEM; statistical significances were assigned by unpaired Student’s *t*-test. hk gene = housekeeping gene; n.s. = not significant. n = 3-8 independent experiments.

To verify that the knockdown of GluN3A did not alter the neural character of the stem cells, transfected cells were analyzed for their expression of a set of NSC and radial markers as well as for their expression of the other NMDAR subunits. Compared to their expression in 46C-derived NSCs transfected with a mixture of scrambled siRNA/scrambled siRNA2, the mRNA expression of NMDARs (GluN1, GluN2A, GluN2B, GluN2C, GluN2D, and GluN3B) did not change significantly when 46C-derived NSCs were transfected with a mixture of GluN3A siRNA1/GluN3A siRNA2 (Fig 6A). Moreover, this also holds true for the mRNA expression of the NSC and radial glia markers GLAST [39], nestin [40], Pax6 [41], and prominin [42] (Fig 6B).

### Transcriptome analysis of 46C-derived NSCs after knockdown of GluN3A

**Fig 7:**
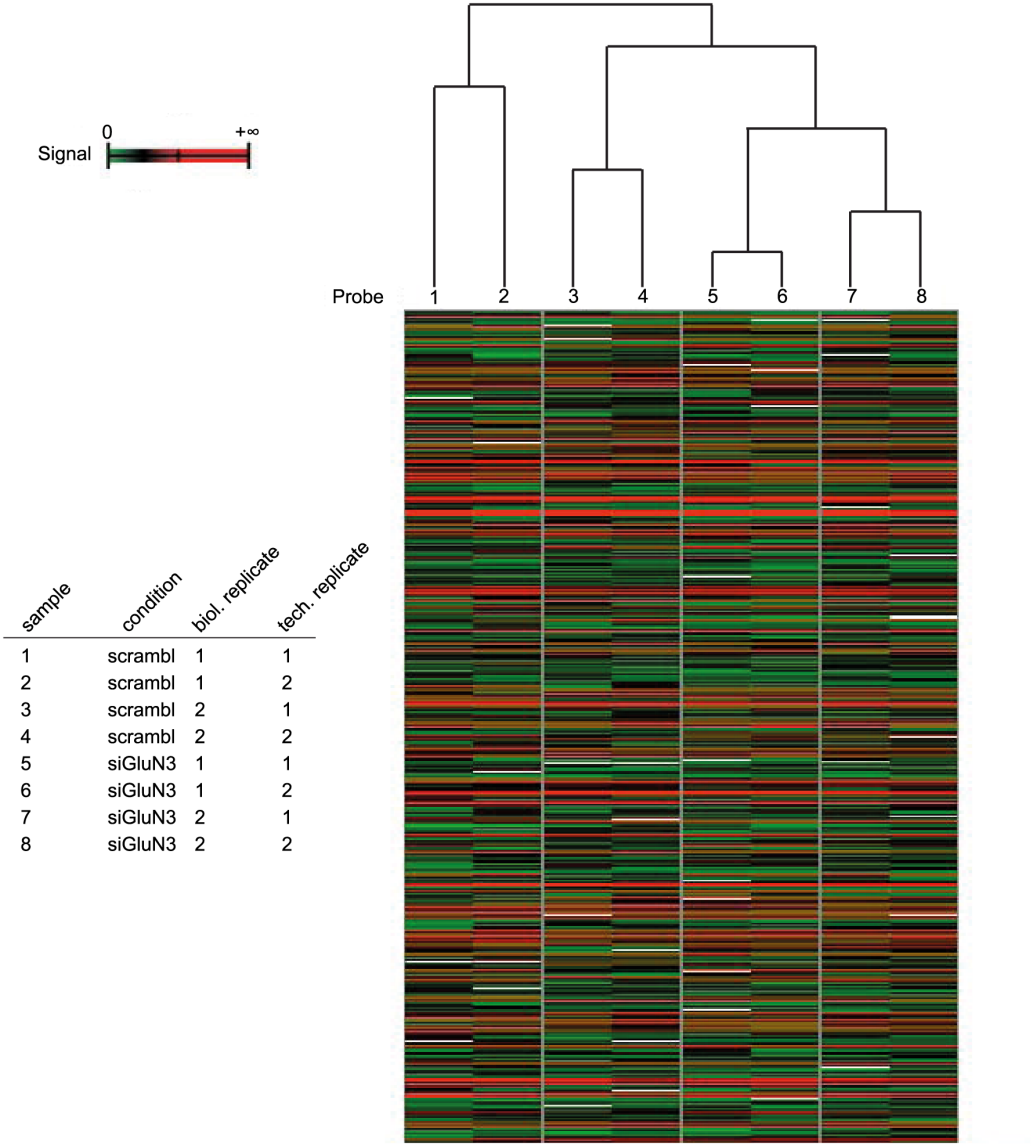
Hierarchical clustering of transcriptome data. Hierarchically clustered biological and technical replicates of the transcriptome data using the Euclidean Distance of the signals as a distance metric. scrambl = scrambled siRNA; siGluN3 = siRNA directed against GluN3A.

In order to identify downstream targets and pathways of GluN3A, the effects of its knockdown were investigated by global gene expression profiling. As initial tests showed that the knockdown of GluN3A mRNA was most efficient when a mixture of both siRNAs was used (see S1 Fig), 46C-derived NSCs were either transfected with scrambled siRNAs1+2 or with siRNAs1+2 directed against GluN3A. Therefore, the transcriptome of two biological replicates (consisting of two technical replicates each) of 46C-derived NSCs upon GluN3A knockdown was compared to the transcriptome of 46 NSCs transfected with the scrambled control siRNAs. Initially, the variability of the generated transcriptome data was monitored by hierarchical clustering of all biological and technical replicates using the Euclidean Distance of the signals as a distance metric (Fig 7), revealing that the technical replicates (sample 1 vs. sample 2, sample 3 vs. sample 4, sample 5 vs. sample 6, and sample 7 vs. sample 8) cluster together and are more closely related to each other than to their respective biological replicates (samples 1+2 vs. samples 3+4, and samples 5+6 vs. samples 7+8, respectively). Furthermore, the biological replicates of 46C-derived NSCs transfected with scrambled siRNAs and the biological replicates of 46C-derived NSCs transfected with GluN3A siRNAs are more closely related within each group (samples 1, 2, 3, 4 for the group of scrambled siRNA and samples 5, 6, 7, 8 for the group of GluN3A siRNA) than to any sample of the respective control group.

**Fig 8:**
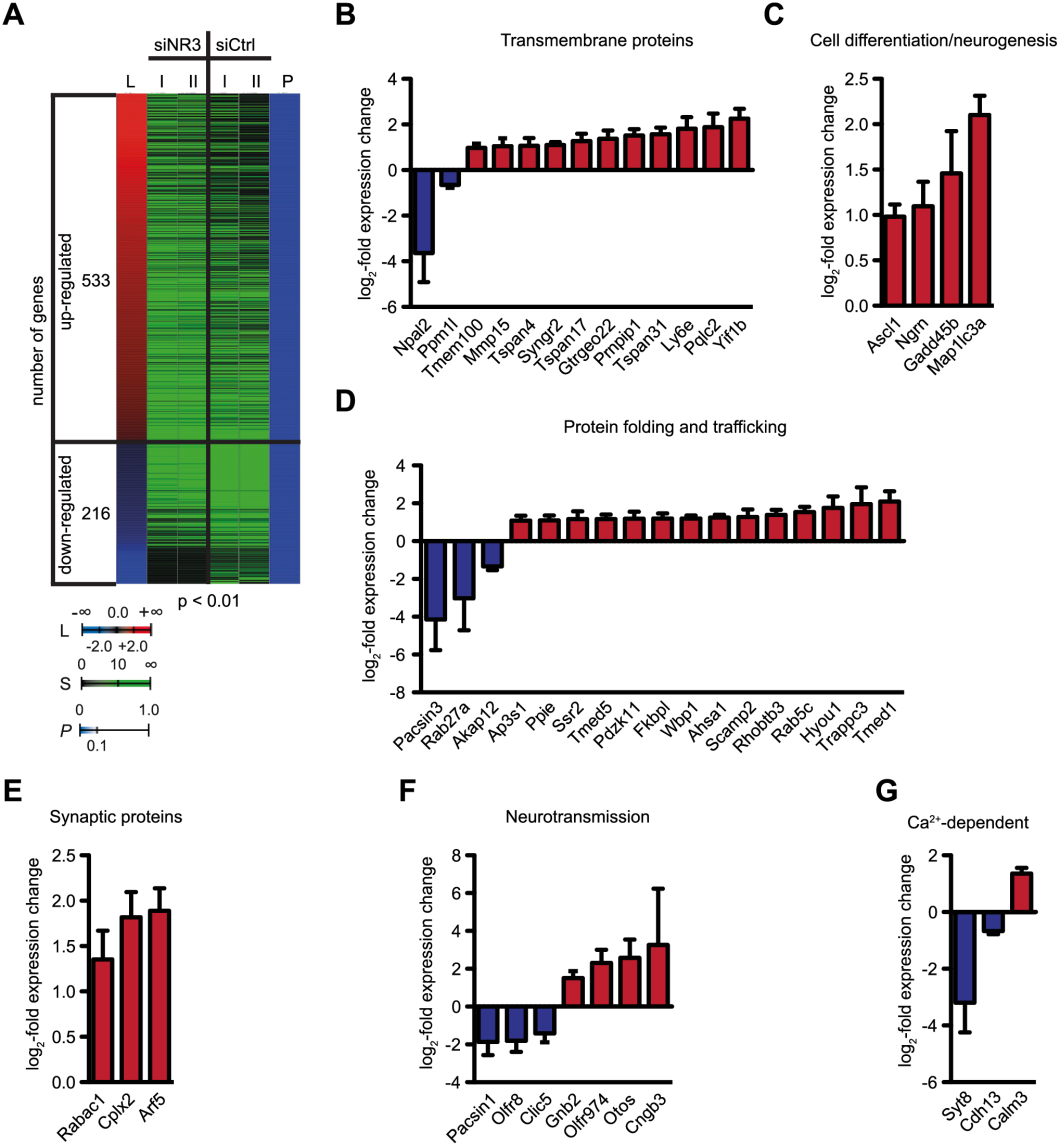
Genes affected by the knockdown of GluN3A in 46C-derived NSCs. **A:** Heatmap of genes statistically significantly (*p < 0.05) differentially expressed in 46C-derived NSCs upon knockdown of GluN3A. GluN3A was knocked down in 46C-derived NSCs using a mixture of GluN3A siRNA1/GluN3A siRNA2 (siNR3), which was compared to a mixture of non-targeting scrambled siRNA1/scrambled siRNA2 (siCtrl) used as a control. Differentially expressed genes were detected using global gene expression profiling. The log_2_-fold changes (L) for statistically significantly up- (red) and down-regulated (blue) genes as well as the signal values (S) for two biological replicates per condition (Roman numerals) and the corresponding P-values (P) are illustrated using a color code. **B to G:** Examples of regulated genes after knockdown of GluN3A in 46C-derived NSCs. The log2-fold change in expression rate is depicted for genes significantly up- and downregulated in 46C-derived NSCs transfected with siRNA against GluN3A in comparison to 46C-derived NSCs transfected with scrambled siRNA. Only genes significantly up- or downregulated (*p < 0.05) are depicted. Red = upregulation; blue = downregulation.

Transfection of 46C-derived NSCs with a mixture of GluN3A siRNA1 and GluN3A siRNA2 significantly (*p* < 0.01) changed the mRNA expression of 749 (2.27%) out of 32 966 murine genes analyzed when compared to 46C-derived NSCs transfected with a mixture of non-targeting scrambled siRNA1/siRNA2 as a control (Fig 8A). Of these genes, 533 were upregulated and 216 were downregulated (Fig 8A). Fig 8B to 8G exemplarily show genes whose expression is affected after knockdown of GluN3A in 46C-derived NSCs. Several of these genes encode for proteins involved in neural function and were clustered into groups based on the functions of their gene products. Among the regulated genes, 17 encode protein folding and trafficking proteins, 15 encode transmembrane proteins, 9 encode proteins involved in cell differentiation and neurogenesis, 8 encode proteins involved in neurotransmission, 5 encode Ca^2+^-dependent proteins, and 4 encode synaptic proteins.

## Discussion

### GluN3A is stably expressed in 46C-derived NSCs

In this study, we show GluN3A mRNA expression in all 46C-derived cell types, with GluN3A transcript expression in 46C-derived NSCs being approximately 70% of the level found in 46C-derived neurons. Moreover, 46C-derived NSCs readily express GluN3A at the protein level. In agreement with our findings, *in vivo*, GluN3A mRNA expression can also be detected from an early stage on. Early GluN3A transcript expression is detected in the embryonic rodent brain [1, 43, 44], is further upregulated during early postnatal development [1, 2, 4, 44] and subsequently declines until adulthood [1, 4, 45]. The protein expression of GluN3A takes a similar course and peaks at P8 [43, 46–50]. Thus, it is conceivable that GluN3A is expressed both at the mRNA and protein levels in 46C-derived NSCs.

As mentioned above, 46C-derived NSCs expressed GluN3A protein, although not at the plasma membrane. We have previously shown that the so-called obligatory NMDAR subunit GluN1 is not yet expressed in 46C-derived NSCs at the protein level [27]. Thus, neither the assembly of triheterotetrameric functional NMDARs nor the expression of functional excitatory glycine receptors composed of GluN1 and GluN3 subunits [51–53] is possible in 46C-derived NSCs. Consistently, we have reported previously that patch clamp analysis of 46C-derived NSCs showed no detectable current after application of NMDA and glycine, or glutamate and glycine [27]. Therefore, we did not attempt to investigate any effect of the knockdown of GluN3A on the survival or proliferation of 46C-derived NSCs. Nevertheless, 46C-derived NSCs can serve as valuable tools to investigate the consequences of a GluN3A knockdown in a system of *in vitro*-differentiated neural cells.

### GluN3A is successfully knocked down in 46C-derived NSCs following synthetic siRNA transfection

In 46C-derived NSCs transfected with either GluN3A siRNA1 or GluN3A siRNA2, GluN3A mRNA levels were significantly decreased to 24% compared to 46C-derived NSCs transfected with scrambled siRNA 1 or 2. This siRNA-mediated knockdown of GluN3A in 46C-derived NSCs was also observed at the protein level. The transfection of scrambled siRNAs did not alter the expression of GluN3A, neither at the mRNA nor at the protein level. Furthermore, the mRNA expression of the other NMDAR subunits and of a set of NSC and radial glia markers was not significantly altered upon knockdown of GluN3A in 46C-derived NSCs. Thus, the transfection of synthetic siRNAs proved to be a suitable and efficient tool to induce a knockdown of GluN3A in 46C-derived NSCs.

### The knockdown of GluN3A affects the mRNA expression of neural genes and iGluR-interacting proteins

Global gene expression profiling revealed that the expression of 749 genes was altered upon knockdown of GluN3A in 46C-derived NSCs. Several of these genes encode for proteins (potentially) involved in neural function, some of which are of particular interest and thus are described in more detail below.

#### Retina-expressed genes

Although GluN3A had initially been cloned from rodent retinal cDNA [4], only a few studies investigated a possible physiological role of GluN3A in the retina. Interestingly, functional Ca^2+^-permeable NMDARs are expressed in rodent and feline retinal ganglion cells (RGCs) prenatally and in the first postnatal days before eye opening and the transmission of visual signals [9, 54, 55]. After retinal maturation, i.e. with the onset of light-evoked signal transmission upon eye opening, functional NMDARs are downregulated. Therefore, it had been suggested that retinal NMDARs mainly play a role in the formation of retinal synaptic connectivity and in synaptic fine-tuning at embryonic and early postnatal stages, but not in the transmission of visual stimuli in the adult retina [54, 55]. Regarding GluN3A, its protein expression can be detected from P3 on in rodent RGCs. It peaks at P10, and then slowly decreases, although GluN3A protein is still expressed in the adult retina [9]. Strikingly, in GluN3A KO mice, NMDA-evoked Ca^2+^ responses are larger than in wildtype (WT) mice, indicating that GluN3A-containing NMDARs are present in WT RGCs [9]. Since GluN3A decreases the Ca^2+^ permeability of NMDARs [2, 4, 5], it is tempting to speculate that GluN3A might play a neuroprotective role in RGCs by decreasing the presumably cytotoxic Ca^2+^ influx through NMDARs [56]. This hypothesis is supported by the observation of an increased NMDA-induced RGC death in GluN3A KO mice compared to WT mice [8].

Upon knockdown of GluN3A in 46C-derived NSCs, the mRNA expression of two retina-expressed genes was altered in comparison to scrambled siRNA-transfected 46C-derived NSCs: Gadd45b (growth arrest and DNA damage protein 45b) was upregulated (275% expression) upon knockdown of GluN3A in 46C-derived NSCs. Interestingly, Gaddb protects RGCs from apoptosis caused by glutamate excitotoxicity [57], and its upregulation might compensate for the lack of the presumably neuroprotective GluN3A subunit. Cngb3 (cyclic nucleotide-gated channel beta 3), was highly upregulated (953% expression). Cyclic nucleotide-gated channels are expressed in rod and cone photoreceptors where they mediate light-evoked signal transmission [58].

#### Small GTPases

Small GTPases are heavily involved in protein trafficking, and several GTPases regulate AMPAR endocytosis [59]. Furthermore, the small GTPases Rheb (Ras homologue enriched in brain) and Rac1 (Ras-related C3 botulinum toxin substrate 1) have been shwon to interact with GluN3A directly or via GTPase-activating proteins [60, 61].

Here, the knockdown of GluN3A in 46C-derived NSCs significantly changed the mRNA expression of GTPases and regulators of GTPases, some of which have been shown before to interact with iGluRs. Rab5c (Ras-related in brain 5c), whose expression was upregulated (289%), regulates AMPAR endocytosis during LTD in an NMDAR-dependent manner [62]. The expression of the small GTPase Arf5 (ADP-ribosylation factor 5; 370%), as well as the expression of the regulatory protein Rabac1 (Rab acceptor 1; 256%) was also highly upregulated after knockdown of GluN3A in 46C-derived NSCs. These proteins could possibly be involved in the trafficking of GluN3A-containing NMDARs.

#### Other genes

NMDARs interact with several cytoskeletal proteins. For example, GluN1 binds to *α*-actinin, thereby linking the NMDAR complex to the actin cytoskeleton [63]. Furthermore, GluN3A is linked to the cytoskeleton via MAP (microtubule-associated protein) 1B and 1S [64, 65]. The knockdown of GluN3A in 46C-derived NSCs results in an up-regulation (429% expression) of MAP1lc3a, one of the light chains of MAP1. Since MAP1B is involved in the control of neurite outgrowth and axon organization during development [66], its upregulation might also interfere with the development of dendritic spines. Calmodulin 3 (calcium-modulated protein), which targets (amongst others) CaMKII (Ca^2+^/calmodulin-dependent kinase II), is upregulated in 46C-derived NSCs upon GluN3A knockdown (256% expression). Consistent with these findings, CaMKII has been previously shown to be upregulated in GluN3A KO mice as well, and the mice show enhanced learning and memory formation, indicating a regulatory role of GluN3A for LTP formation in WT mice [67]. Strikingly, another upregulated gene upon GluN3A knockdown in 46C-derived NSCs was Pdzk11 (PDZ domain-containing 11; 227% expression). Pdzk11 contains a single PDZ domain, thus providing a putative physical link to NMDARs, and interacts with the plasma membrane Ca^2+^-ATPase (PMCA) [68], which in turn was suggested to have a neuroprotective function [69]. Since GluN3A was also suggested to play a neuroprotective role [3, 6, 8, 9], it is tempting to speculate that its knockdown leads to an increased interaction of Pdzk11 with conventional diheteromeric NMDARs and also to an increased interaction with PMCA to compensate for the loss of GluN3A.

Three tetraspanins were upregulated upon GluN3A knockdown, namely Tspan4, Tspan17, and Tspan31 (209%, 242%, and 297% expression, respectively). Members of the transmembrane family of tetraspanins have been shown to interact with iGluRs in previous studies [70]. Finally, PACSIN1 and PACSIN3 (protein kinase C and casein kinase substrate in neurons protein 3) were drastically downregulated upon GluN3A knockdown (27% and 6% expression, respectively). Although PACSIN1 interacts with GluN3A through the C-terminal domain of GluN3A, to date no interaction of GluN3A with PACSIN3 has been observed [12]. Nevertheless, the strong downregulation of PACSIN3 might indicate an interaction with the NMDAR complex through other means, e.g. with another NMDAR subunit or an associated protein.

Notably, in 2000, the existence of a large NMDAR multiprotein complex (NRC) has been discovered via proteomic analysis of mouse forebrain extracts [71]. This receptor-adhesion molecule complex is composed of 77 proteins, among these cell adhesion proteins, cytoskeletal proteins, and GTPases, some of which are also significantly regulated in the present microarray study (e.g. calmodulin and MAP2B). However, whereas GluN1, GluN2A, and GluN2B were detected in the NRC, GluN3A was not [71]. Thus, it is conceivable that triheteromeric NMDARs interact with slightly different or additional proteins than NMDAR complexes without GluN3A.

**Fig 9:**
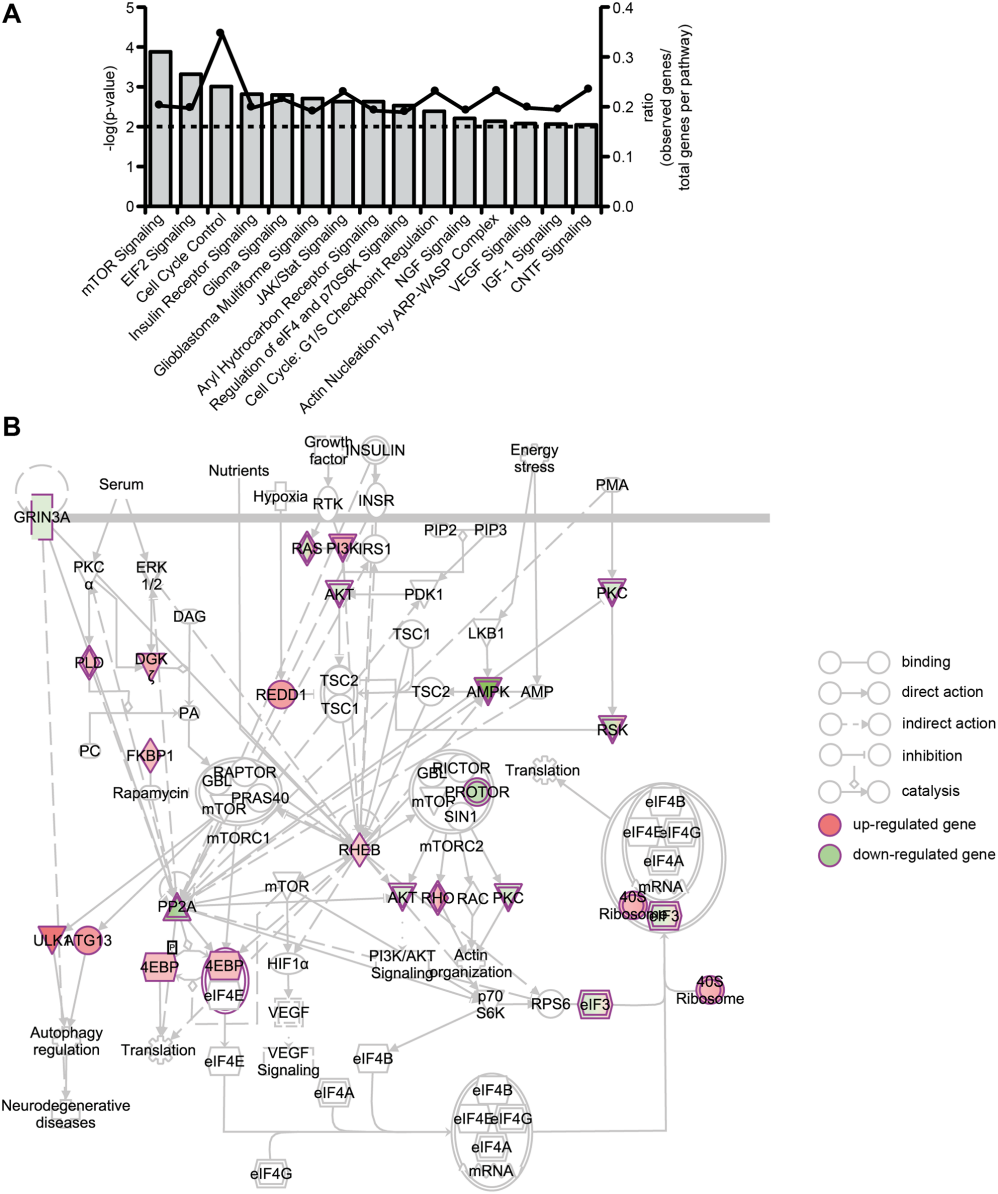
Canonical pathways affected by the knockdown of GluN3A in 46C-derived NSCs. **A:** Canonical pathway enrichment studies for GluN3A target genes. Canonical pathways statistically significantly (**p < 0.01) enriched for genes that were statistically significantly (*p < 0.05) affected by GluN3A knockdown in 46C-derived NSCs were identified using Ingenuity Pathway Analysis software. The –log(P-value) (grey bars) as well as the ratio (black dots) are illustrated. The P-value threshold (*p* < 0.01) is shown as a dotted line. **B:** The complete mTOR pathway (Ingenuity Pathway Analysis) is illustrated. Connecting arrows are described below the image. Genes statistically significantly (*p* < 0.05) up-regulated are shown in red, those down-regulated are shown in green.

Based on the set of genes statistically significantly (*p* < 0.05) regulated by the knockdown of GluN3A, we performed canonical pathway enrichment studies in order to identify cellular pathways significantly impacted (*p* < 0.01) in this condition (Fig 9A). The observed pathways cover key cellular functions such as cell growth, proliferation, motility, survival, cell cycle regulation, and translation regulation (Fig 9A). The most significantly affected pathway is the mechanistic target of rapamycin (mTOR) pathway, involving two direct interaction partners of GluN3A: Ras homolog enriched in brain (Rheb) [61] and protein phosphatase 2 (PP2A) [47] (Fig 9B). PP2A activity has been shown to be increased upon GluN3A binding [47] and the interaction with Rheb has been previously suggested to affect mTOR signaling, whose dysregulation has been linked to alterations in spine density [61]. While some of the enriched pathways (e.g. mTOR or eIF4 and P70S6K signaling) involve PP2A directly, others depend on the PP2A targets or interaction partners protein kinase C (PKC) [72, 73] (e.g. Insulin receptor signaling, glioma signaling, VEGF signaling, NGF signaling, and IGF-1 signaling), Janus kinase 2 (JAK2) [74] (e.g. JAK/Stat signaling, CNTF signaling, and IGF-1 signaling) or cell division cycle 6 (Cdc6) [75] (e.g. cell cycle regulation). Thus, our findings suggest a possible functional role of GluN3A within the cellular pathways of its direct interaction partners PP2A and Rheb.

To summarize, in the present study, the NMDAR subunit GluN3A was efficiently knocked down at the mRNA as well as at the protein level in 46C-derived NSCs via an siRNA approach. Following transcriptome analysis, it was revealed that GluN3A affects the mRNA expression of a number of neural genes, whose corresponding gene products in part have been previously shown to interact with NMDARs. Canonical pathway enrichment studies identified key cellular pathways involving the GluN3A interaction partners Rheb and PP2A to be targeted by the knockdown of GluN3A. Though the mechanisms still need to be investigated in detail in future studies, our results point to a regulatory function of this non-conventional NMDAR subunit within biological pathways involved in cell growth, proliferation, and motility as well as cell survival.

## Supporting information

S1 Fig siRNA-mediated knockdown of GluN3A mRNA in 46C-derived NSCs. 46C-derived NSCs were either transfected with a mixture of scrambled siRNAs1+2 or with a mixture of siRNAs1+2 directed against GluN3A. **A:** qRT-PCRs were performed to analyse the knockdown of GluN3A at the mRNA level. GluN3A was significantly downregulated upon transfection with the mixture of siRNAs1+2 against GluN3A. There were no statistically significant differences in GluN3A expression between non-transfected NSCs and NSCs transfected with the mixture of scrambled siRNAs1+2 or scrambled siRNA2. Data represent means +/- SEM; statistical significances were assigned by unpaired Student’s *t*-test. ***p < 0.001. n = 3 independent experiments.
(TIF)
