# Supplementary figures and images for: The siRNA-mediated knockdown of GluN3A in 46C-derived neural stem cells affects mRNA expression levels of neural genes, including known iGluR interactors
Source: PLoS One. 2018 Feb 13;13(2):e0192242. doi: 10.1371/journal.pone.0192242 (PMC5811004; doi:10.1371/journal.pone.0192242)

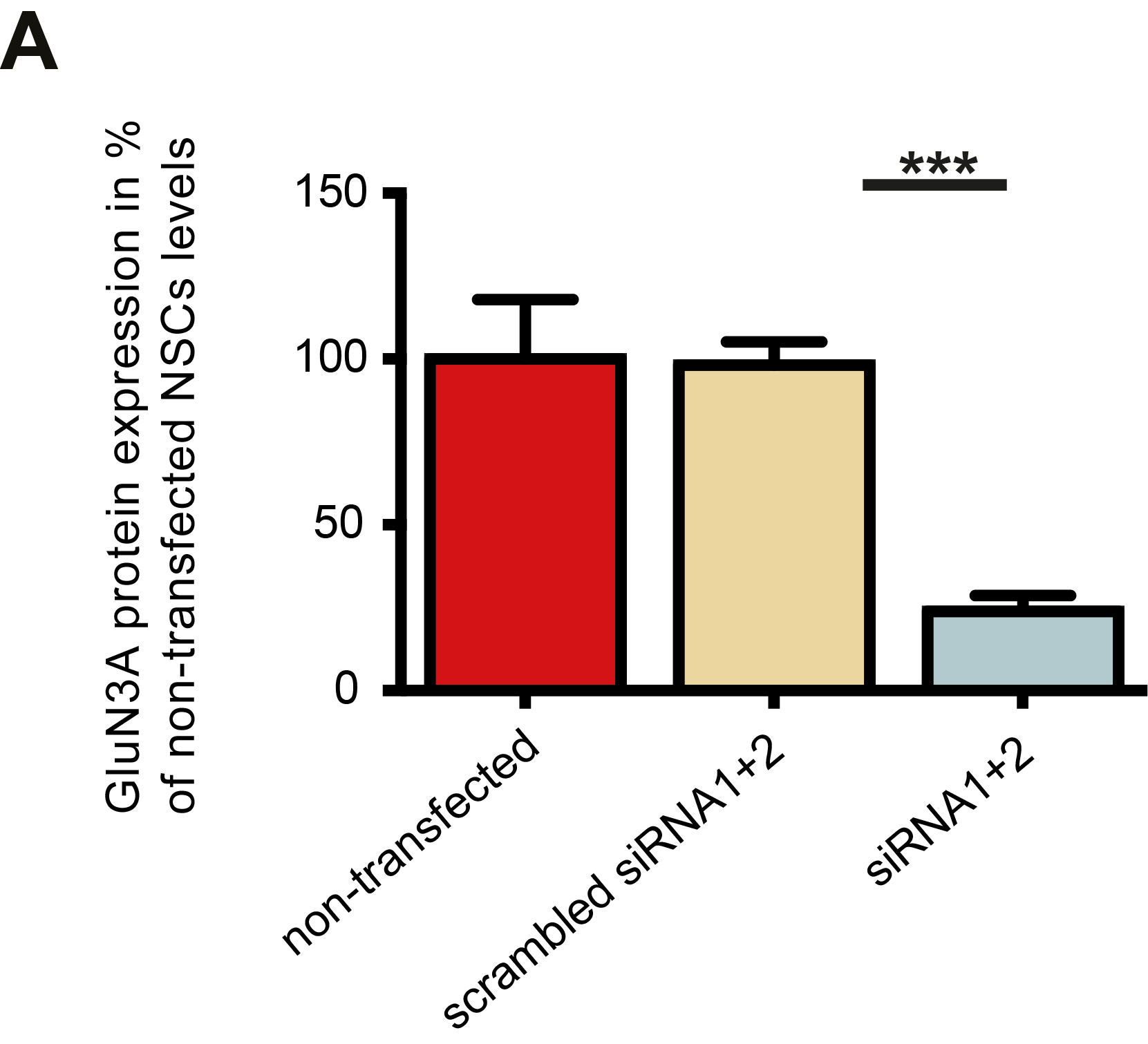

Supplement: S1 Fig — 46C-derived NSCs were either transfected with a mixture of scrambled siRNAs1+2 or with a mixture of siRNAs1+2 directed against GluN3A. A: qRT-PCRs were performed to analyse the knockdown of GluN3A at the mRNA level. GluN3A was significantly downregulated upon transfection with the mixture of siRNAs1+2 against GluN3A. There were no statistically significant differences in GluN3A expression between non-transfected NSCs and NSCs transfected with the mixture of scrambled siRNAs1+2 or scrambled siRNA2. Data represent means +/- SEM; statistical significances were assigned by unpaired Student’s t-test. ***p < 0.001. n = 3 independent experiments. (TIF) [file pone.0192242.s001.tif]
